# Development of Drug Delivery Systems Based on Layered Hydroxides for Nanomedicine

**DOI:** 10.3390/ijms15057750

**Published:** 2014-05-05

**Authors:** Farahnaz Barahuie, Mohd Zobir Hussein, Sharida Fakurazi, Zulkarnain Zainal

**Affiliations:** 1 Materials Synthesis and Characterization Laboratory, Institute of Advanced Technology (ITMA), Universiti Putra Malaysia, UPM, Serdang 43400, Malaysia; E-Mails: farahB2424@yahoo.com (F.B.); zulkar@putra.upm.edu.my (Z.Z.); 2 Laboratory of Vaccines and Immunotherapeutics, Institute of Bioscience, Universiti Putra Malaysia, UPM, Serdang 43400, Malaysia; E-Mail: sharida@medic.upm.edu.my; 3 Department of Human Anatomy, Faculty of Medicine and Health Sciences, Universiti Putra Malaysia, UPM, Serdang 43400, Malaysia

**Keywords:** layered hydroxides, nanomedicine, drug delivery, nanocomposite, nanocarrier, LDH, ZLH, cytotoxicity assay

## Abstract

Layered hydroxides (LHs) have recently fascinated researchers due to their wide application in various fields. These inorganic nanoparticles, with excellent features as nanocarriers in drug delivery systems, have the potential to play an important role in healthcare. Owing to their outstanding ion-exchange capacity, many organic pharmaceutical drugs have been intercalated into the interlayer galleries of LHs and, consequently, novel nanodrugs or smart drugs may revolutionize in the treatment of diseases. Layered hydroxides, as green nanoreservoirs with sustained drug release and cell targeting properties hold great promise of improving health and prolonging life.

## 1. Introduction

The delivery of bioactive and therapeutic molecules is one of the most interesting areas of research with increasing growth and importance in medicine. Conventional therapeutic systems suffer from rapid release of drugs, with no control over release rate and fluctuation in drug levels in the blood stream and other target organs, enzymatic degradation, poor water solubility, and the necessity to use high drug doses, which may cause many adverse effects. Therefore, the focus on the development of effective reservoirs for efficient delivery of drugs has been the priority of today’s medical research [1–3].

One of the most important and fascinating advancement in science is the development of nanomaterials. Nanomaterials are materials with at least one dimension in the nanoscale range, which gives them unusual physical and chemical features, including quantum effect, high reactivity, and high surface area to volume ratio. Although, nanomaterials can be synthesized in one, two, or three dimensions, two dimensional nanosheets have deeply fascinated scientists because of their unique interaction properties.

Nanocomposites are hydrid materials with different phases in which one of the phases has at least one dimension at the nanoscale size regime [4]. Layered nanocomposites are formed by insertion of a guest anion into the interlayer region of the inorganic interlayers without changes in the layered structure, and this is called “intercalation”. Organic-inorganic nanocomposites are very inventive structures that provide an unlimited set of new nanocomposites due to the combinations of the organic and inorganic components [5].

Layered hydroxides (LHs) are inorganic materials that can be used as hosts to construct organic-inorganic nanocomposites. LHs are composed of nanolayers with two-dimensional infinite layers with nanoscale thickness and offer extensive applications in various areas. These host-guest layered solids can be classified as layered double hydroxides (LDH) and layered hydroxide salts (LHS).

## 2. Structure of Layered Hydroxides

**Figure 1.**
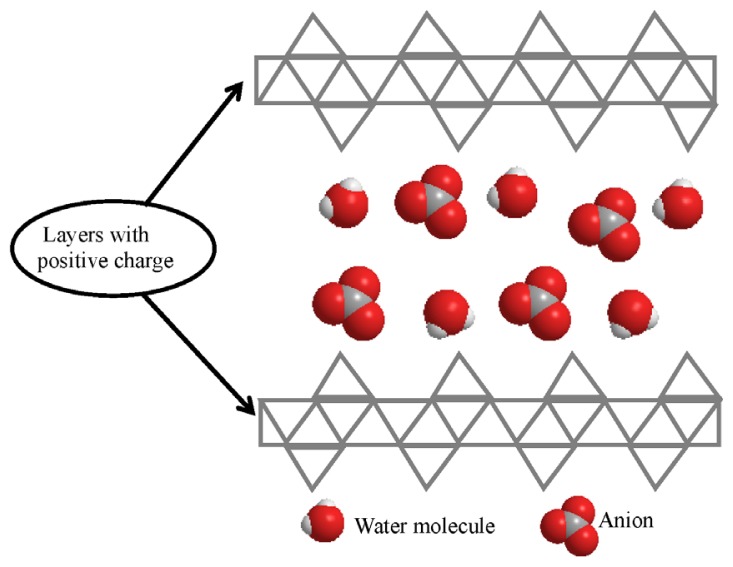
Structure of Layered hydroxides.

Layered hydroxides: layered hydroxide salts and layered double hydroxides are derived from a brucite layered structure, which is a naturally occurring magnesium hydroxide and was first discovered in 1824. The brucite structure is composed of a centering magnesium ion between six octahedrally arranged hydroxide ions. Each hydroxide ion is bonded to three magnesium atoms [6]. Layered double hydroxides can be represented by the general formula, [*M*^2+^_1−_*_x_M*^3+^*_x_*(OH)_2_]*^x^*^+^(*A^n^*^−^)*_x_*_/_*_n_*·*m*H_2_O, where *M*^2+^ is a divalent cation, such as Mg^2+^, Zn^2+^, Ni^2+^, Mn^2+^, Co^2+^, *etc.*, *M*^3+^ is a trivalent cation, such as Al^3+^, Fe^3+^, Cr^3+^, Co^3+^, *etc.*, and *A^n^*^−^ denotes exchangeable organic or inorganic anions with charge (*n*−), such as NO_3_^−^, CO_3_^2−^, Cl^−^, SO_4_^2−^, OH^−^, which compensates the excess positive charge due to the isomorphical divalent/trivalent substitution in the interlayer region, while m represents the amount of water that is located in the interlayers (Figure 1) [7].

The general formula of layered hydroxide salts is *M*^2+^(OH)_2−_*_x_*(*A^n^*^−^)*_x_*_/_*_n_*·*m*H_2_O, where *M*^2+^ represents a metallic cation, such as Zn^2+^, Mg^2+^, Co^2+^, Ni^2+^, Cu^2+^, Cd^2+^, *etc.*, and *A* is a counter ion with (*n*−) charge (Figure 1) [8]. Zinc layered hydroxide (ZLH) is a layered hydroxide salt that can be classified into two groups. Group I contains the layers with octahedral coordinated zinc atoms to six hydroxide groups, with an empirical formula of Zn_2_(OH)_2_(NO_3_)_2_·2H_2_O, where the nitrate anions are directly coordinated to the zinc. Group II can be divided into two; Types II_a_ and II_b_ with empirical formula, Zn_5_(OH)_8_(NO_3_)_2_·2H_2_O and Zn_5_(OH)_8_(NO_3_)_2_, respectively. Although, in both Types, 1/4 of octahedral zinc cations are located in tetrahedral sites above and below each empty octahedron and hydroxide ions shared with the octahedral sheet occupy the three vertices of the tetrahedron, the water molecules in Type II_a_ occupy the apex and nitrate ions are located between the layers surrounded by water molecules. However, in Type II_b,_ nitrate ions occupy the apex and are directly coordinated with the zinc tetrahedron [9].

## 3. Synthesis Methods of Layered Hydroxides

### 3.1. Synthesis Methods of Layered Double Hydroxides

There are several methods of synthesis that can be adopted, *i.e.*, co-precipitation, ion exchange, urea hydrolysis, and reconstruction/rehydration method.

#### 3.1.1. Co-Precipitation Method

The co-precipitation technique is the most commonly used and simplest method. A solution containing the anion guest is added to an aqueous solution of two different metals that are used as precursors. This is followed by drop-wise addition of an alkaline solution to the mixture under vigorous stirring and a nitrogen atmosphere until a final pH of 7–10. The mixture is aged at 70 °C for 18 h and the resultant slurry is then filtered, washed with water, and finally dried in an oven at 60 °C. This method produces large quantities of nanocomposite and the packing density of the interlayer anion is diverse due to variable *M*^+2^/*M*^+3^ ratios. Furthermore, a wide diversity of anions can be incorporated between the layers. However, there is a significantly higher uptake of carbon dioxide and incorporation of unwanted hydroxide anions from the reaction mixture [7,10,11].

##### Precipitation at High Supersaturation

The precipitation at high supersaturation method requires the addition of a mixed metals salt solution to an alkaline solution that contains the interlayer anions. The materials that are synthesized by this method usually have low crystallinity, and therefore thermal treatment or aging is used after co-precipitation to increase the crystallinity of the materials [12].

##### Precipitation at Low Supersaturation

Precipitation at low supersaturation is accomplished by slow addition of mixed solutions of divalent and trivalent cation metal salts to an aqueous solution of the interlayer anion. The alkaline solution is added simultaneously to maintain the pH at selected value to lead to co-precipitation of the two metallic salts. This method gives LDHs with high crystallinity and allows control of the molar ratio, *R* = *M*^2+^/*M*^3+^.

#### 3.1.2. Ion Exchange Method

When the metal cations or intercalated anions are unstable, the co-precipitation method is not applicable for the encapsulation procedure. Therefore, in these cases, the ion exchange method is useful. A solution containing anionic guest is added to 50 mL of an aqueous solution containing the LDH. The pH of the reaction mixture is kept at 7–10 by simultaneous addition of an alkaline solution and the suspension formed is magnetically stirred vigorously for 3 h. The slurry is aged at 70 °C for 18 h and then filtered, washed with decarbonated water, and dried in an oven at 60 °C. In this method, the guest anions are exchanged with the anion present in the interlayer space [13].

The ion-exchange process may be affected by some factors namely:

- Incoming anion affinity.
  The ion exchange process will be alleviated if the incoming anion has a higher charge and smaller size (smaller ionic radius) [14].
- Exchange media
  The organic solvent favors the exchange by organic anions, whereas the aqueous medium favors the exchange by inorganic anions [15].
- pH value
  pH value affects the interaction between the LDH layers and interlayer conjugated base anions. At low pH, this interaction is weak because the LDH is not stable in acidic media (lower than pH 4) and this will result in the fact that the LDH dissolves.
- Chemical composition
  The hydration state and charge density have an effect on the ion-exchange process and the charge density and hydration state of the LDH’s interlayer can be affected by the chemical composition of the layers in LDH [16].

#### 3.1.3. Hydrothermal Method

The hydrothermal method is usually used during the preparation or intercalation process to control particle size and size distribution in order to produce LDHs with uniform size and high crystallinity [17].

#### 3.1.4. Urea Hydrolysis Method

Urea has some excellent features and can be used as a precipitation agent. The LDH prepared by this method has homogeneous sizes and platelet-like primary particles with well-defined hexagonal shapes and good crystal quality [18].

#### 3.1.5. Reconstruction/Rehydration Method

This method involves calcination and regeneration of LDHs. In LDH calcination, removal of the water interlayer and anions, and dehydroxylation of the layer occurs, resulting in the formation of a metal oxide. In addition, exposing the metal oxide mixture to water and anions leads to regeneration of the layer structure. At the end, anions intercalate into the interlayer gallery and water reforms the hydroxyl layers [19].

### 3.2. Synthesis Methods of Layered Hydroxide Salts

Layered hydroxide salts can be synthesized by urea hydrolysis, solid state reaction, co-precipitation, and hydrolysis of salts and oxides techniques.

#### 3.2.1. Urea Hydrolysis

LHS can be prepared by this method according to the equations given below. Urea hydrolysis is the source of the hydroxide group. The LHS formation occurs in the presence of nitrate or chloride metal salt solutions as a consequence of an increase in the OH^−^ concentration (Equation (4)).

(1) $${\text{NH}}_{2}-\text{CO}-{\text{NH}}_{2}\to {\text{NH}}_{3}+\text{NHCO}$$

(2) $$\text{HNCO}+2{\text{H}}_{2}\text{O}\to {\text{NH}}_{4}\text{OH}+{\text{CO}}_{2}$$

(3) $${\text{NH}}_{3}+{\text{H}}^{+}\to {{\text{NH}}_{4}}^{+}$$

(4) $${\text{M}}^{2+}{\text{A}}_{y}+2-x\;{\text{OH}}^{-}\to \text{M}{\left(\text{OH}\right)}_{2-x}{\left({\text{A}}^{m-}\right)}_{x/m}+y-x\;{\text{A}}^{m-}$$

As shown in Equation (2), the carbonate species is produced by further reaction of carbon dioxide with water. This method cannot be used for the synthesis of LHS-NO_3_ or LHS-Cl because the formation of carbonate species in which carbonate has a higher affinity towards inorganic interlayers compared to nitrate or chloride anions subsequently prevents intercalation [20].

#### 3.2.2. Solid State Reaction

LHS-NO_3_ in the presence of urea can also be synthesized by this method in which the hydroxide ions are generated by urea. In this method, the proper amounts of hydrated metal nitrate salt are mixed with urea and the reaction is accomplished under solid state conditions, so that the amount of water is reduced to a minimum. It is then followed by heating of the resulting material, washing, and filtering [21].

#### 3.2.3. Precipitation Method

In this method, LHS is precipitated by the addition of an alkaline solution to the aqueous solution. In addition, addition of an excess alkaline solution can cause solubilization of the metal oxide as a result of LHS hydrolysis. Therefore, controlling the molar ratio of OH^−^/*M*^+^ is very important in this precipitation technique [22].

#### 3.2.4. Hydrolysis of Salts and Oxides

The reaction can be carried out between aqueous solutions of divalent metal salts and metal oxide. When the divalent metal cation salts contain the same metal as the oxide, LHS is formed. However, when the metal salts are different from the oxide, the product is DHS [23,24].

## 4. Characterization of Layered Hydroxides

A broad range of analytical techniques are used to characterize layered hydroxides. Powder X-ray diffraction is used to determine purity, crystallinity, and the basal spacing of the LDHs by scrutinizing recorded X-ray diffraction patterns. Fourier transform infrared (FTIR) spectroscopy is used to identify the functional groups and chemical bands, because each functional group has its own specific wavenumbers and characteristic absorption peaks. Therefore, FTIR can be used as a supporting technique to prove that intercalation has occurred instead of absorption. The carbon, hydrogen, nitrogen, and sulfur (CHNS) analyses are used to determine the percentage of these elements in the samples and at the same time prove the presence of guest anions between the layers and intercalation episode. The chemical composition of the LDHs is analyzed for metal ions by inductively coupled plasma atomic emission spectrometry. The thermal stability of LDHs is measured using thermogravimetric and differential thermogravimetric analyses (TGA/DTG) while the surface characterization of the LDH is carried out using surface area analysis. A field emission scanning electron microscope is used to determine the surface morphology of the samples. Optical properties and the controlled release studies are accomplished using an ultraviolet-visible spectrophotometer. Transmission electron microscopy (TEM) is used for particle size determination of layered hydroxides. For TEM studies, the specimens are first prepared by adding a known amount of the LHs to 5 mL of a suitable solvent followed by sonication to obtain well dispersed samples. A drop of the solution is placed on a grid, dried in air, and further analyzed.

## 5. Applications of Layered Hydroxides

Layered hydroxides, with outstanding physico-chemical properties and their tailor-made behavior offer extensive applications in different areas, as listed below (Table 1).

## 6. Layered Hydroxides in Drug Delivery Systems

**Figure 2.**
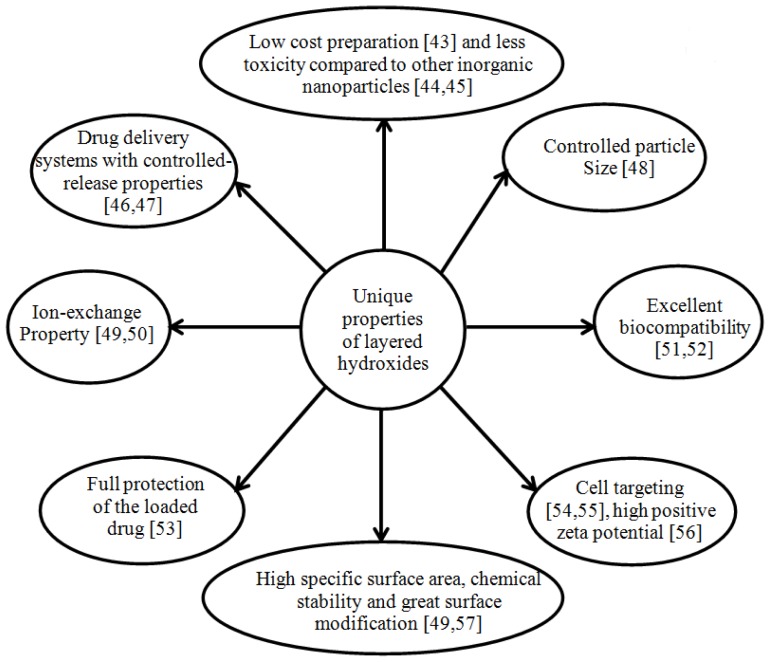
Advantages of layered hydroxides for drug delivery systems [43–57].

Recently, LDHs and zinc layered hydroxide have gained considerable attention due to their unique features (Figure 2) as nanocarriers in drug delivery systems.

These materials are synthesized from biocompatible metal elements and have pH-dependent solubility, so that they can easily be decomposed in the acidic biological environment. Their anion exchange property allows loading of various drugs into the interlayer lamellae of LHs, which leads to modification of the charge density of the internal and external surfaces, resulting in greater chemical stability, cell targeting function, and high surface area.

The rate-controlled drug delivery property resulted in the reduction of drug concentration fluctuations and maintains drug concentration at the desired level for longer periods of time, decreases adverse effects, and reduces the number of doses and therapy duration, which leads to more effective treatment. In addition, the high positive zeta potential of LHs is due to the stronger adhesion to the negatively charged cell membrane. Moreover, the degradation and anion formation of these two dimensional materials in liposomes (pH 4.5–5) allow them to leave the cell through the ion tunnels of the cell membrane.

The blood biocompatibility properties of layered hydroxides make them unique injectable drug nanoreseviors. Previous *in vivo* studies of anticancer drugs, such as methotrexate, podophyllotoxin, and 5-fluorouracil intercalated into layered hydroxides, showed that the intercalated drugs from nanocomposites are released slowly in a controlled manner into blood fluids (pH 7.4) and they also exhibited good blood clearance compared to free drugs [54,58,59].

### 6.1. Anticancer Drug Therapy Using Layered Hydroxides

#### 6.1.1. Protocatechuic Acid

Protocatechuic acid (PA), an active anticancer drug, has been encapsulated in the Mg/Al-LDH interlayer by ion-exchange and direct coprecipitation methods. Protocatechuic acid has unique pharmacological properties, such as anticancer, antimutagenic, antioxidant, cardioprotective, anti-inflammatory, and chemopreventive properties [60]. As a result of the intercalation process, the thermal stability of the intercalated protocatechuic acid was significantly enhanced compared with free protocatechuic acid, and the protocatechuic acid anion was accommodated as a monolayer with an angle from the *z*-axis of 8° and 15° in PANE (protocatechuic acid-Mg/Al nanocomposite synthesized by ion-exchange method) and PAND (protocatechuic acid-Mg/Al nanocomposite synthesized by direct method), respectively. In addition, the release of protocatechuic acid from the nanocomposites at pH 7.4, 5.3, and 4.8 was much slower than its release from a physical mixture, which was fast and completed within 10 min. The release of PA from the PANE nanocomposite occurred within 1000, 3894, and 7500 min at pH 4.8, 5.3, and 7.4 in phosphate buffer solution, respectively, and from PAND nanocomposite was around 900, 1349, and 4000 min at pH 4.8, 5.3, and 7.4 phosphate buffer solution, respectively. Therefore, the release of protocatechuic acid from nanocomposites was sustained and makes them a promising candidate for controlled-drug release applications. Furthermore, release kinetic studies indicated that the pseudo-second order model (Table 2) could better describe the release kinetic processes of protocatechuic acid from PANE at pH 7.4 and 4.8, and from PAND at pH 7.4, while the kinetic release of protocatechuic acid from PAND and PANE in a pH 5.8 environment could be described by the parabolic diffusion model and pseudo-first order (Table 2), respectively. On the other hand PAND at pH 4.8 obeyed pseudo-first order kinetic release [61].

#### 6.1.2. Etoposide

Etoposide (VP16) is an antitumor drug [84], which is widely applied in the treatment of germ cell tumors, childhood malignancies, small cell lung carcinoma, gastric cancer cells, and hematologic malignancies. However, this drug suffers from poor water solubility and very short plasma half-life that require high dose administration and causes drug resistance and side effects. The study revealed a new delivery for etoposide. The drug was intercalated into the Mg/Al-layered double hydroxide matrix using a two-step procedure.

The average diameter of the nanohybrid particle size was 62.5 and the zeta potential was 20.5 mV upon intercalation. The nanohybrid showed a good buffer effect at low pH environment. Comparison of the release profiles of etoposide from the nanohybrid that took place within 390 min in both pH 4.6 and 7.4 media compared with its release from a physical mixture that was completed within 20 min, revealed that the release of VP16 from the nanohybrid took place in a controlled manner and the resulting nanohybrid was deemed suitable for a controlled-release formulation. Parabolic diffusion and modified Freundlich models (Table 2) were more satisfactory for describing the release kinetics of etoposide from the nanohybrid at pH 7.4, while the release mechanism at pH 4.8 could be described by the parabolic diffusion model (Table 2). Furthermore, thermogravimetry and differential thermogravimetry analyses indicated that the thermal stability of VP16-LDH was markedly increased [75].

#### 6.1.3. Methotrexate

Mg/Al-LDH was successfully employed to intercalate methotrexate (MTX), which is commonly used to treat different cancers. Cancer therapy with MTX suffers from fluctuations in drug concentration in the blood stream as a result of its short half-life, which necessitates frequent drug administration that leads to adverse effects. The *in-situ* ion exchange route was used for nanocomposite synthesis. The thermal stability of the incorporated MTX was remarkably higher than the free molecule. The particle size of the LDH-MTX nanocomposite was in the range of 50–800 nm with a zeta potential of 36.3 mV, which demonstrated moderate stability. The MTX release from the nanocomposite at pH 7.4 occurred in two stages: at the beginning, around 50% of MTX was released in 7 h, followed by a relatively slower stage of 90% for the second 95 h and the release was completed in 190 h. The MTX release from the nanohybrids in pH 7.4 phosphate buffer saline followed relatively slow and fitting by the Ritger-Peppas model (Table 2) showed that the mass transfer process occurred through a combination of crystal dissolution, ion-exchange, and diffusion [78].

Methotrexate (MTX) anticancer drug was also encapsulated into the Zn/Al-layered double hydroxide (LDH) by an anion exchange technique. Particle size of the resulting nanohybrid was in the range of 100–300 nm and the intercalated methotrexate was thermally more stable than unbound MTX.

The release of methotrexate from the MTX-Zn/Al-LDH nanohybrid in pH 7.4 phosphate buffer solution was around 90% in 24 h and the release was completed within 48 h. In addition, the Ritger-Peppas kinetics model (Table 2) resulted in the best fit for the release of MTX from the nanocomposite and showed that the drug release occurred via diffusion [79].

#### 6.1.4. Camptothecin

Camptothecin is nonionic anticancer drug with poor water solubility and has been broadly applied in the treatment of human lung, ovarian, breast, pancreas and stomach cancers [85]. For new delivery purposes of camptothecin, a drug-inorganic clay hybrid with camptothecin (CPT) intercalated into a Mg/Al-layered double hydroxide (LDH) has been made by a reconstruction method for the formation of the CPT-Mg/Al-LDH nanohybrid. The water solubility of the CPT-Mg/Al-LDH nanohybrid was three times higher than the free drug’s solubility. The release of CPT from the nanohybrid was sustained and significantly slower than the release of camptothecin and the pristine Mg/Al-layered double hydroxide at pH 7.2 and 4.8. Furthermore, the release of CPT from the nanohybrid at pH 7.2 was slower than the release at pH 4.8, due to different release mechanisms. At pH 4.8, the mechanism possibly occurred through the dissolution of LDH layers, while at pH 7.2, diffusion was the main mechanism involved. Moreover, the release of CPT from the nanohybrid might follow by pseudo-second order kinetic model (Table 2) [47].

#### 6.1.5. Gallic Acid

Gallic acid (GA), an anticarcinogenic agent [86], has been incorporated into the Zn/Al-layered double hydroxide matrix using the ion exchange method. GA has interesting pharmacological properties, including anticarcinogenic, anti-inflammatory, anti-mutagenic, and antimicrobial properties. The loading of GA in the nanocomposite was around 42.2% (*w*/*w*). The thermal stability of the intercalated GA was significantly enhanced compared to the free drug. The surface area of the nanocomposite increased compared to Zn/Al-LDH upon the intercalation process. The cumulative release of GA from the nanocomposite occurred in a controlled manner. The release of the drug was fast for the first 500 min and then followed by a slow release in carbonate aqueous solutions (0.002, 0.005, and 0.01 M). The initial rapid release was due to the fast ion exchange that was caused by the high concentration of carbonate ions in the solution. The kinetic study showed that the release of GA from the nanocomposite satisfactorily followed pseudo-first order kinetics (Table 2) [62].

GA has also been intercalated into calcined zinc layered hydroxide (ZLH) layers by the direct method. The thermal decomposition of GA increased from 263 to 554 °C as a result of the intercalation of GA into the interlayers of ZLH. It was found that the intercalated GA was thermally more stable than free GA. In addition, the surface area of the nanohybrid increased upon intercalation, and the loading of GA in the nanohybrid was around 52.36% (*w*/*w*) [87].

#### 6.1.6. 5-Fluorouracil

The chemotherapeutic agent 5-fluorouracil (5-FU) has been encapsulated into the interlayer gallery of a Zn/Al-layered double hydroxide nanocarrier via the ion exchange method. This chemotherapeutic agent is broadly applied in cancer treatment, especially in colorectal cancer via intravenous administration [88]. However, cancer therapy with 5-FU caused several adverse effects, such as cardiac, dermatological, neural, and gastrointestinal toxic side-effects as a result of the short half-life and the required high dose administration. The release of 5-FU from the 5-FU/cyclodextrin-Zn/Al-LDH nanocomposite was shown to be sustained and the Zn/Al-layered double hydroxide was able to prolong the release time. Moreover, the release mechanism of 5-FU from the 5-FU/cyclodextrin-Zn/Al-LDH nanocomposite followed Korsmeyer-Peppas kinetics (Table 2), which is associated with drug diffusional control [83].

#### 6.1.7. Fenbufen

The antitumor agent fenbufen has been incorporated into Mg/Al-LDH and Mg/Al/Fe-LDH by different techniques. Fenbufen is a drug with antitumor and anti-inflammatory properties [89], but displays some side-effects with regard to the central nervous system and gastrointestinal tract. The solubility and thermal stability of fenbufen increased as a result of intercalation compared to free fenbufen. The resulting nanohybrids contained between 31% and 44% (*w*/*w*) fenbufen. The release of fenbufen from the nanohybrids occurred in a controlled manner and slower than the release from a physical mixture. Furthermore, the drug release mechanism fitted the Weibull kinetic model (Table 2) well [82].

#### 6.1.8. Ellagic Acid

Ellagic acid (EA) has been incorporated into the interlayer of ZLH using the direct reaction with zinc oxide in a nonaqueous environment. The resulting nanohybrid was a mesoporous type material and its basal spacing was 10.4 Å. The ellagic acid molecule between the layers of the ZLH was orientated in a monolayer fashion with an angle of 22.5° from *z*-axis.

The cumulative release of ellagic acid from the nanohybrid was lower than from a physical mixture and occurred in a controlled manner (69% in Na_2_CO_3_ and 94% in Na_3_PO_4_ aqueous solution). Furthermore, pseudo-second order kinetics (Table 2) was established for the release mechanism of ellagic acid from the nanohybrid [65].

#### 6.1.9. Hippuric Acid

Hippuric acid has been intercalated into a zinc layered hydroxide nanovehicle via a simple direct technique. The resulting nanohybrid contained 38.75% (*w*/*w*) of guest hippuric acid and the encapsulated hippuric acid was thermally more stable than the unbounded drug. In addition, the surface area of the nanohybrid increased significantly. The release of hippuric acid from the nanohybrid was much slower than the release from a physical mixture. The release of the drug from the nanohybrid was sustained with 99.8% within 21 h, 56.4% within 63 h and 95.7% within 72 h at pH 4.8 and 7.4 phosphate buffer solution and an aqueous solution of 0.08 M Na_2_CO_3_, respectively.

The release of guest from the nanohybrid at pH 7.4 was lower than at pH 4.8 due to different release mechanisms. The release at pH 4.8 occurred by dissolution of ZLH layers, because ZLH is unstable in acidic media, while in pH 7.4, the release took place through the ion-exchange between the hippuric acid anions and anions in the buffer solution. Furthermore, the release profile in a Na_2_CO_3_ solution was governed by pseudo-second order kinetics (Table 2), while first order kinetics (Table 2) showed the best fit for release at pH 7.4. Interestingly, the release profile in pH 4.8 did not obey any kinetic model [63].

#### 6.1.10. Prednisone

Prednisone-cholate ion micelles have been intercalated in Mg/Al-LDH by the direct co-precipitation technique. Prednisone is an anticancer drug [90] with poor water solubility that can be applied in the treatment of different cancers.

The intercalation process resulted in increased water solubility of prednisone compared with free prednisone. The release profiles of the nanocomposite at pH 4.8 and 6.8 in phosphate buffer solution was completed within 105 min, while the release rate at pH 7.6 media was slower compared with pH 4.8 and 6.8, and was completed in 175 min. In addition, the release of prednisone from the nanocomposite might be controlled through dissolution of LDH layers, ion-exchange between prednisone and anions in the phosphate buffer solution, and disaggregation of the cholate micelle. The release mechanism of prednisone from the nanocomposite at pH 4.8 and 6.8 followed first-order kinetics (Table 2), while the Bhaskar equation (Table 2) could satisfactorily describe the release profile at pH 7.6 [64].

#### 6.1.11. Carnosine and Gallic Acid

Carnosine and GA have been incorporated into Mg/Al layered double hydroxide lamellae using direct and ion exchange methods. Carnosine and GA show an excellent range of pharmacological activities, such as antioxidant, anti-inflammatory, anticancer, antivirus, and anti-allergic properties [86,91]. However, carnosine can be hydrolyzed rapidly in blood, while GA has a short half-life. Both drugs can be oxidized under moderate conditions and have pH sensitivities; all these factors lead to a significant reduction in their biological functionality.

Carnosine and GA nanohybrids showed amazing antioxidant activity, as deduced from their 2,2-diphenyl-1-picrylhydrazy (DPPH) radical scavenging capacities, which were 83.9% for GA-Mg/Al-LDH and 95.9% for carnosine-Mg/Al-LDH in 870 min. The release of both drugs from the nanohybrids at pH 7.4 phosphate buffer solution consisted of two stages. The first stage was fast, but without burst effect so that this step provides the therapeutic dose. A slow stage, which was second stage and able to maintain the drug concentration constant for long period of time. The percent release of carnosine from the nanohybrid was higher than GA with 71.1% and 61.1%, respectively. Furthermore, in the release profile of both drugs, the first stage controlled the release rate via external surface diffusion through ion exchange and the release mechanism of this stage was governed by parabolic diffusion kinetics (Table 2) [76].

#### 6.1.12. Chlorogenic Acid

Chlorogenic acid (CA) is an anticancer agent [92] and has been encapsulated into zinc layered hydroxide interlayers by a simple and direct reaction. Chlorogenic acid (CA) is a naturally occurring organic compound, which is widely present in many plants. It is well-known for its unique biological activities, including antioxidant activity, anti-HIV, anti-inflammatory, anti-carcinogenic, and antitumor activities. In addition, CA is stable at room temperature and can be decomposed at 60°.

The intercalation process resulted in an improved thermal stability of CA compared with the free drug. The loading of chlorogenate in the nanhybrid was around 46.1% (*w*/*w*). The release of chlorogenate from the nanohybrid was around 99% in 161 h at pH 7.4 compared to around 88% in 80 h at pH 4.8 phosphate-buffered saline (PBS) and the release dynamics was described well by pseudo-second order kinetics (Table 2). Furthermore, the nanohybrid showed the properties of a mesoporous type of material [66].

### 6.2. Anti-Hypertensive Drug Therapy by Layered Hydroxides

#### 6.2.1. Perindopril Erbumine

The incorporation of perindopril erbumine, an antihypertensive drug [93], into Zn/Al-LDH has been studied to show that perindopril erbumine-Zn/Al-LDH nanohybids can be used as an effective drug delivery system. The intercalated perindopril erbumine was thermally more stable than its free counterpart. The intercalation process resulted in expansion of the basal spacing of the nanohybrid to 19.9 Å for PZAC (perindopril-Zn/Al nanocomposite synthesized by direct method) and 21.7 Å for PZAE (perindopril-Zn/Al nanocomposite synthesized by ion exchange method). The loading of perindopril into the nanohybrids was around 37.2% for PZAE and around 33.4% for PZAC, and the nanohybrids were shown to be mesoporous type materials. In addition, perindopril was accommodated in monolayer mode in PZAC, while it was oriented in bilayer fashion in PZAE interlayers. The drug release from the nanohybrids showed controlled release and the release at pH 7.4 was much lower than at pH 4.8, because of different release mechanisms. Interestingly, the release rate of perindopril from PZAE was faster than from PZAC. Pseudo-second order kinetics (Table 2) provided the best fit for the release of perindopril from the nanohybrids [67].

The intercalation of perindopril into Mg/Al-LDH using ion exchange has also been investigated. The intercalated perindopril was thermally more stable than its free counterpart and the loading percentage of the drug into the nanohybrid was around 36.5% (*w*/*w*). In addition, the basal spacing of the perindopril-Mg/Al-LDH nanohybrid was expanded to 21.98 Å. The perindopril release from the nanohybrid indicated sustained release and the release rate of perindopril from the nanohybrid at pH 7.4 (100% release in 5000 min) was remarkably lower than at pH 4.8 (100% release in 1000 min), possibly due to the different release mechanisms. Furthermore, the mechanism of perindopril release from the nanocomposite could be well described by pseudo-second order kinetics (Table 2) [68].

#### 6.2.2. Enalapril, Lisinopril, Captopril, and Ramipril

The incorporation of enalapril, lisinopril, captopril, and ramipril, all antihypertensive drugs [94–96], into a Zn/Al-layered double hydroxide matrix through ion-exchange or direct methods has been studied before. The basal spacing of the nanohybrids was expanded to 21.61 Å for captopril-Zn/Al-LDH, 12.46 Å for enalapril-Zn/Al-LDH, 26.56 Å for ramipril-Zn/Al-LDH, and 20.98 Å for lisinopril-Zn/Al-LDH. The captopril and ramipril anions were accommodated in an alternately tilted bilayer arrangement, along the long axis orientation in a proper angle between the layers of Captopril-Zn/Al-LDH and ramipril-Zn/Al-LDH nanohybrids. However, lisinopril and enalapril anions were arranged in alternately vertical monolayer mode, along the short axis for enalapril and along the long axis for lisinopril. In addition, the intercalated enalapril, lisinopril, captopril, and ramipril were significantly more stable than their free molecules upon intercalation.

The release profile of the drugs from the nanocomposites was much slower than from a physical mixture at pH 4.25 and 7.45 phosphate buffer solutions. The drug release from the physical mixture at pH 4.25 and 7.45 occurred in 12 and 17 min, respectively. On the other hand, the release of enalapril, lisinopril, captopril, and ramipril from the nanocomposites at pH 4.25 was around 81.3% in 92 min, 76.8% in 92 min, 80.6% in 62 min and 82.8% in 72 min, respectively. The release of enalapril, lisinopril, captopril, and ramipril from the nanocomposites at pH 7.45 took place in 232, 232, 172 and 192 min, respectively, and the release rate was slower than at pH 4.25, which was due to different release mechanisms. The Zn/Al-LDH was not stable at pH 4.25 and release occurred through the dissolution of layers, while Zn/Al-LDH was more stable in a pH 7.45 environment and the release possibly occurred through ion-exchange between the drug anions in the interlayer and phosphate anions in the buffer solution. The Higuchi square root (Table 2) was determined to be the best fit for the release mechanism of enalapril, lisinopril, captopril, and ramipril from their nanohybrids [81].

### 6.3. Anti-Inflamatory Drug Therapy by Layered Hydroxides

#### 6.3.1. Salicylic Acid

The anti-inflammatory agent, salicylic acid has been encapsulated in zinc layered hydroxide interlayers using a direct reaction for the new delivery system. The intercalated salicylic acid anion, salicylate, was thermally more stable than its free molecule. The resulting intercalated compound showed mesoporous material properties and a basal spacing of 23.9 Å as a result of the fact that salicylate intercalated in a monolayer arrangement with an angle of 57° between the interlayer spaces of ZLH. The nanohybrid contained around 29.66% (*w*/*w*) of salicylic acid anions. The surface area of the nanocomposite remarkably increased to around 49 m^2^/g upon intercalation [97].

#### 6.3.2. Diclofenac

The intercalation of diclofenac, an anti-inflammatory drug, into Zn/Al-layered double hydroxide through ion-exchange has been studied. The thermal analysis of the nanohybrid indicated that the decomposition of intercalated diclofenac occurred at around 500 °C. In addition, the diclofenac-Zn/Al-LDH nanocomposite contained about 41.8% of diclofenac anions, while the basal spacing of the resulting nanohybrid was expanded to 22.5 Å. The release profiles of diclofenac from the nanocomposite at pH 7.5 and 7.0 PBS and pH 7.0 PBS containing sodium chloride and sodium carbonate indicated that the percentage of diclofenac release at pH 7.5 PBS (90% after 24 h) was higher than at pH 7.0 PBS and pH 7.0 PBS containing sodium chloride and sodium carbonate (both 72% release after 24 h). The mechanism of diclofenac release could be described satisfactorily by the Bhaskar equation (Table 2) [80].

#### 6.3.3. Sodium Indomethacin

The anti-inflammatory drug sodium indomethacin has been hybridized into the gallery space of Mg/Al-LDH using direct co-precipitation and ion-exchange routes. Mg/Al-LDHs, which are used for the intercalation of sodium indomethacin via the ion-exchange method, were synthesized at two different aging times: LDHS (short aging time) and LDHL (long aging time) with LDHL showing a higher crystallinity than LDHS.

The basal spacing of the three nanocomposites was expanded to 25.21, 19.39–25.96 and 20.24 Å with sodium indomethacin loading of 48.40, 7.83 and 13.98 wt % for the nanocomposite prepared by direct co-precipitation, ion-exchange at 20 min and ion-exchange method at 20 h, respectively.

Moreover, as a result of the intercalation process, the water solubility of intercalated sodium indomethacin significantly increased compared to its free molecule [98].

### 6.4. Anti-Histamine Drug Therapy by Layered Hydroxides

#### Cetirizine

Cetirizine is an antihistamine drug [99] and has been intercalated into Mg/Al-LDH and Zn/Al-LDH interlamellae via the ion exchange route in order to design new delivery systems for cetirizine.

In allergic reactions and in the case of tissue injury, release of histamine can occur. Cetirizine dihydrochloride, as a second-generation antihistamine, blocks the H1 receptor, thereby preventing histamine release. The thermal stability of intercalated cetirizine was enhanced compared to free cetirizine upon incorporation of cetirizine into the LDHs. The intercalation process resulted in expansion of basal spacing of nanohybrids to 31.2 Å for CTMAN (cetirizine-Mg/Al nanocomposite) and 31.9 Å for CTZAN (cetirizine-Zn/Al nanocomposite), and cetirizine was accommodated in a tilted bilayer mode in both nanohybrids. The resulting nanohybrids contained 60.7% and 57.2% of cetirizine in CTMAN and CTZAN, respectively. The percentage release of cetirizine from the CTMAN nanhybrid was around 96.3% in 2980 min and 97.8% in 750 min at pH 7.4 and 4.8, respectively. Conversely, the percentage release from the CTZAN nanohybrid was around 95.6% in 600 min and 96% in 600 min at pH 7.4 and 4.8, respectively. The drug release from the nanohybrids was sustained and the release rate of cetirizine from the nanohybrids at pH 7.4 was significantly slower than at pH 4.8 due to different release mechanisms. Furthermore, the release kinetics of cetirizine from the nanocomposites was fitted satisfactorily by pseudo-second order kinetics (Table 2) [69].

The intercalation of cetirizine into zinc layered hydroxide for the formation of a new nanocomposite was also accomplished using a simple and direct technique. The resulting nanohybrid contained 49.4% (*w*/*w*) of cetirizine and the basal spacing of the cetirizine-ZLH nanohybrid was expanded to 33.9 Å upon intercalation. The guest, cetirizine was thermally more stable than its free unbound counterpart and the nanohybrid showed mesoporous properties. In addition, cetirizine was arranged in a horizontal bilayer mode between the layers of ZLH. The release of cetirizine from the nanohybrid occurred in a controlled manner with around 96% in 80 h at pH 7.4 and around 97% in 73 h at pH 4.8 of phosphate buffer solution, and the release dynamics was described well by pseudo-second order kinetics (Table 2) [70].

### 6.5. Sunscreen Drug Therapy by Layered Hydroxides

#### 6.5.1. Cinnamic Acid

The intercalation of an efficient UVA and UVB absorber [100], cinnamic acid, into the zinc layered hydroxide layers by a direct technique has been studied. The loading of the cinnamic acid anions into the nanocomposite was around 40.4% *w*/*w* and the basal spacing of the cinnamate-ZLH nanocomposite was expanded to 23.9 Å as a result of the intercalation process. In addition, cinnamic acid anions were accommodated in a bilayer fashion between the zinc layered hydroxide inorganic interlayers. The resulting nanohybrid had the unique ability to absorb UVA and UVB and showed mesoporous material properties. The release of cinnamic acid anions from the nanohybrid occurred in a controlled manner with 47.34% in 6 days, 21.07% in 4.4 days, and 57.16% in 13 h in deionized water, 0.5 mol/L NaCl solution, and pH 5.5 phosphate buffer, respectively. Furthermore, the release mechanism could be described satisfactorily by pseudo-second order kinetics (Table 2) [71].

#### 6.5.2. Caffeic Acid

The intercalation of caffeic acid, a UV shielding molecule, within the gallery of zinc layered hydroxide using the ion exchange route has been reported. The caffeic acid-ZLH nanocomposite contained 47.4% of caffeic acid. In addition, the basal spacing of the nanohybrid was expanded to 10.4 Å as a result of a bilayer arrangement with an angle of 74° caffeic acid anions between the layers of the ZLH matrix. Furthermore, the UV shielding property of the nanohybrid was remarkably enhanced upon intercalation compared to free caffeic acid [101].

The extracted caffeic acid from *Eupatorium Adenophorum* has been incorporated into the gallery space of Mg/Al-LDH via ion-exchange method. The basal spacing of the caffeic acid nanocomposite increased to 22.54 Å upon intercalation [102].

### 6.6. Anti-Tuberculosis Drug Therapy by Layered Hydroxides

#### 4-Amino Salicylic Acid

The 4-amino salicylic acid (4-ASA), an anti-tuberculosis drug [103], has been hybridized with zinc layered hydroxide using the direct method. This anti-tuberculosis drug has been used previously for the treatment of tuberculosis. However, with classical treatment regimens, 4-ASA shows many adverse effects, including diarrhea, nausea, abdominal cramps, anorexia, vomiting, and epigastric distress. Therefore, the authors of this study developed a new delivery system for 4-ASA. The nanohybrid contained 16.19% of 4-ASA with a basal spacing of 24.0 Å, which showed that the 4-ASA anions were arranged as a bilayer between the zinc-layered hydroxide interlayers. In addition, the resulting nanohybrid showed the property of a mesoporous type material. The release of 4-ASA from the nanocomposite was sustained, with 100% release in 1330 min and 92% release in 8540 min at pH 4.8 and 7.4, respectively. Moreover, the release of 4-ASA from the nanocomposite could be described well by pseudo-second order kinetics (Table 2) [72].

### 6.7. Anti-Parkinsonian Drug Therapy by Layered Hydroxides

#### Levodopa

The intercalation of the anti-Parkinsonian active agent [104], l-3-(3,4-dihydroxyphenyl) alanine (levodopa), into the zinc-layered double hydroxide as the host via the co-precipitation method has been studied. The thermal stability of the intercalated levodopa was significantly enhanced, as a result of the intercalation process and the percentage of drug loading was around 16% (*w*/*w*) in the nanocomposite. The basal spacing of levodopa-Zn/Al-layered double hydroxide nanocomposite was expanded to 10.9 Å as a result of monolayer arrangement of levodopa between the zinc aluminum layered double hydroxide interlayers. The release profile of levodopa from the nanocomposite occurred in a sustained manner with 74% release in 2400 min at pH 4.8 and 76% release in 8600 min at pH 7.4 of phosphate buffer solution. Furthermore, pseudo-second order kinetics (Table 2) was shown to be the best fit for the release mechanism of levodopa from the nanocomposite [73].

Levodopa has also been encapsulated into a Mg/Al-LDH matrix using two successive ion-exchange procedures. As a result of the intercalation process, the intercalated levodopa was thermally and stereo-chemically more stable than unbound levodopa, and showed high resistance to racemization. The resulting nanohybrid contained about 75% of levodopa anions and its basal spacing was expanded to 13.12 nm as a result of a vertical monolayer arrangement of levodopa anions between the layers of Mg/Al-LDH. The release profile of levodopa from the nanocomposite at pH 7.6 and 6.4 of phosphate buffered solution occurred in a controlled manner with 65% and 92% drug release, respectively [105].

### 6.8. Vitamins Storage and Delivery by Layered Hydroxides

#### 6.8.1. Folic Acid

Folic acid, the supplemental form of B vitamin complex, has been incorporated into a Mg/Zn/Al-layered double hydroxide matrix via the co-precipitation route.

The intercalated organic moiety in the nanohybrid was thermally more stable than free folic acid. In addition, the nanohybrid contained 45.2% (*w*/*w*) folic acid anions and the basal spacing of the nanohybrid was around 1.42 nm, and the folic acid anion was accommodated in a tilted monolayer with an angle of 60°.

The folic acid-LDH nanocomposite exhibited good buffering properties at low pH (pH 4), which was due to the excellent antacid property of the layered double hydroxide. The release of folic acid from the nanocomposite occurred in a controlled manner with 75% in 250 min and the release mechanism was governed satisfactorily by the parabolic diffusion model (Table 2) [77].

Folic acid has also been encapsulated into the gallery of Mg/Al-layered double hydroxide using the ion exchange and direct co-precipitation methods for this new delivery system. The thermal stability of the intercalated folic acid was remarkably enhanced upon intercalation. The basal spacing of the nanohybrids was expanded to15.3 and 16.0 Å in nanocomposites produced via direct and ion exchange methods, respectively. As a result, the folic acid anions were oriented in a tilted longitudinal monolayer with an angle of 60° in the nanocomposite produced via the co-precipitation method and 58° in the nanocomposite produced via the ion exchange method. In addition, the loading of folic acid in the nanohybrids was around 19.32% for direct preparation and 17.89% for ion exchange preparation. Furthermore, the resulting nanohybrids exhibited a good buffer property in the pH range of 3–4 [106].

#### 6.8.2. Ascorbic Acid

Vitamin C (ascorbic acid) can easily be oxidized in the presence of dioxygen. It can also react easily with metal ions under neutral and alkaline conditions. In addition, in biosystems, vitamins can easily be decomposed by enzymes. Therefore, vitamin C has been hybridized into the Mg/Fe-LDH and Zn/Fe-LDH matrix using ion exchange. The basal spacing of the nanocomposites was 10.8 Å for Zn/Fe-LDH and 11.5 Å for Mg/Fe-LDH, and the intercalated vitamin C was more stable than free vitamin C. The adsorption isotherms of the nanohybrids followed the Langmuir model. The pseudo-first order kinetics model (Table 2) could satisfactorily describe vitamin C’s adsorption by the layered double hydroxides. Moreover, release of vitamin C from the vitamin C-Mg/Fe-LDH nanocomposite was sustained and slower than from the vitamin C-Zn/Fe-LDH nanocomposite, which indicated that Mg/Fe-LDH is the appropriate nanocarrier for vitamin C [74].

### 6.9. Antibiotic Drug Therapy by Layered Hydroxides

#### 6.9.1. Ciprofloxacin

The intercalation of ciprofloxacin (CFX) into Zn/Al-LDH layers has been accomplished by using ion-exchange and direct methods. Ciprofloxacin is an antibiotic with strong antibacterial properties and is widely applied in the treatment of gram-positive cocci and gram-negative bacteria. However, the gastro-intestinal irritation in ciprofloxacin therapy was observed. The thermal stability of intercalated ciprofloxacin was markedly enhanced compared to its free molecule. The basal spacing of nanohybrids was 20.61 and 20.37 Ǻ for the nanocomposites prepared by the direct and ion exchange method, respectively, and the ciprofloxacin anion was arranged in a monolayer fashion in the gallery space of the Zn/Al-LDH nanocarrier [107].

#### 6.9.2. Paracetamol

Paracetamol has been intercalated into the interlayers of a Mg/Al-LDH mixed oxide by utilizing a rehydration reaction. Paracetamol is water soluble without forming paracetamol ions. As a result of the intercalation process, the thermal stability of the paracetamol nanocomposite was markedly enhanced and the basal spacing of the resulting nanocomposite increased to 20 Å. In addition, the paracetamol molecules were accommodated in a rather disordered arrangement between the layers of layered double hydroxide.

The *in vitro* release study of the paracetamol nanocomposite showed that release was sustained and slower than release from tablets containing the powdered pharmaceutical in distilled water (pH 6.7), phosphate buffer solution (pH 7.5), and 0.1 M HCl solution (pH 1.0). The release of paracetamol from the tablets was fast in all media with near equal release rates and was not affected by the pH. The release of paracetamol from the nanocomposite in distilled water showed the lowest release rate. However, the drug release rate from the nanocomposite in phosphate buffer solution at pH 7.4 was slightly higher, while the release in 0.1 M HCl solution at pH 1.0 was significantly faster than the release in phosphate buffer solution (pH 7.5) and distilled water (pH 6.7). Moreover, the release of paracetamol from the nanocomposites was governed by pseudo-second-order kinetics (Table 2) [19].

#### 6.9.3. Chloramphenicol

Diclofenac, ketoprofen, and chloramphenicol have all been hybridized in Mg/Al-LDH, Zn/Al-LDH and Mg/Zn/Al-LDH through direct co-precipitation techniques.

As a result of the intercalation, the basal spacing of the nanohybrids was expanded and was 11.8, 12, and 11.5 Å for chloramphenicol-Mg/Zn/Al-LDH, chloramphenicol-Zn/Al-LDH, and chloramphenicol-Mg/Al-LDH nanocomposite, respectively, 23.6, 22.0, and 23.3 Å for diclofenac-Mg/Zn/Al-LDH, diclofenac-Zn/Al-LDH, and diclofenac-Mg/Al-LDH nanocomposite, respectively, and 24.7, 23.9, and 21.5 Å for ketoprofen-Mg/Zn/Al-LDH, ketoprofen -Zn/Al-LDH, and ketoprofen -Mg/Al-LDH nanocomposite, respectively. Furthermore, the chloramphenicol anions were arranged in a bilayer and ketoprofen and diclofenac anions were accommodated in a slightly tilted bilayer, perpendicular between the layers of the layered double hydroxides. Moreover, the thermal stability of the nanohybrids significantly increased compared to the free molecules [108].

## 7. Toxicology Studies of Layered Hydroxides and Layered Hydroxide Nanodelivery Systems

The cytotoxicity of an agent is characterized by its destructive action on certain cells. Cell death usually occurs through two processes: apoptosis and necrosis. Necrosis is the death of a cell caused by external physical or chemical factors, which causes loss of cell membrane integrity. Conversely, apoptosis, which is also called programmed cell death or cellular suicide, takes place under normal physiological conditions. The changes and death of cells in apoptosis are indicated by membrane blebbing, caspase activation, cell shrinking, nuclear fragmentation and chromatin condensation [109].

Cytotoxicity assays provide predictive evidence on compound safety and specify the toxicity degree of compounds through the measurement of different parameters, such as mitochondrial activity or cellular adenosine triphosphate (ATP) levels, cell membrane integrity by measuring the uptake of a fluorescent dye or measuring lactate dehydrogenase leakage in the extracellular space, and cell numbers by determination of cellular protein or DNA [110,111].

**Figure 3.**
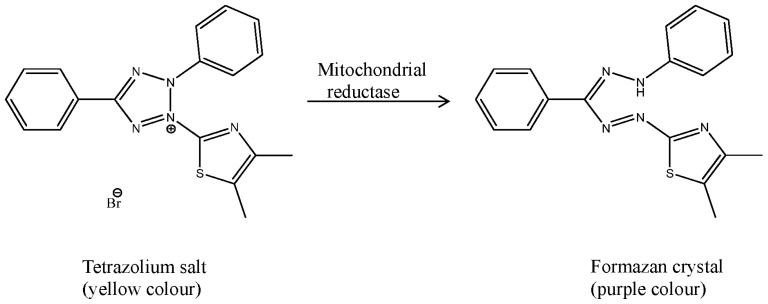
The formation of formazan crystals from the tetrazolium salt.

The colorimetric methyl tetrazolium (MTT) assay measures mitochondrial dehydrogenase enzyme activity in living cells through the reduction of the yellow water-soluble tetrazolium bromide salt to the purple formazan, which is not soluble in water (Figure 3) [112]. The results in this assay are evaluated by plotting the cell viability percentage (*y*-axes) against the concentration of drug (*x*-axes).

**Figure 4.**
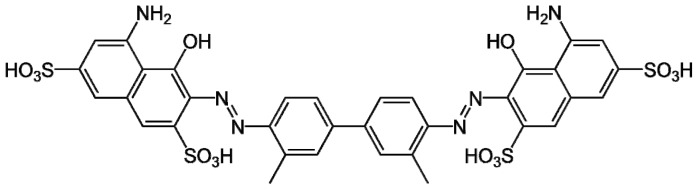
Molecular structure of trypan blue.

The trypan blue (Figure 4) exclusion assay is a viability test, which is also called dye exclusion test. It is used to determine the toxicity of compounds in cells. It colorized dead cells are subsequently observed by their blue color under a microscope. The viable cells do not show blue color, because the dye does not readily traverse the intact plasma membrane of live cells. The percentage of viable cells is calculated by determining the stained cells relative to the total number of cells [113].

The cytotoxicity of layered hydroxides and their nanohybrids have been investigated in different cell lines. Layered hydroxides have extensively been studied for medical and biological applications compared to other inorganic nanoparticles. The MTT and trypan blue assays showed that layered double hydroxides (Mg/Al-LDH and Zn/Al-LDH) did not affect cell proliferation and viability up to 500 μg/mL. On the other hand, long exposure times (72 h) and high doses of 250–500 μg/mL caused plasma membrane damage, inflammation, and oxidative stress, which could be measured through the released of lactate dehydrogenase into extracellular medium. Furthermore, the Zn/Al-LDH showed slightly higher toxicity than Mg/Al-LDH [114].

**Figure 5.**
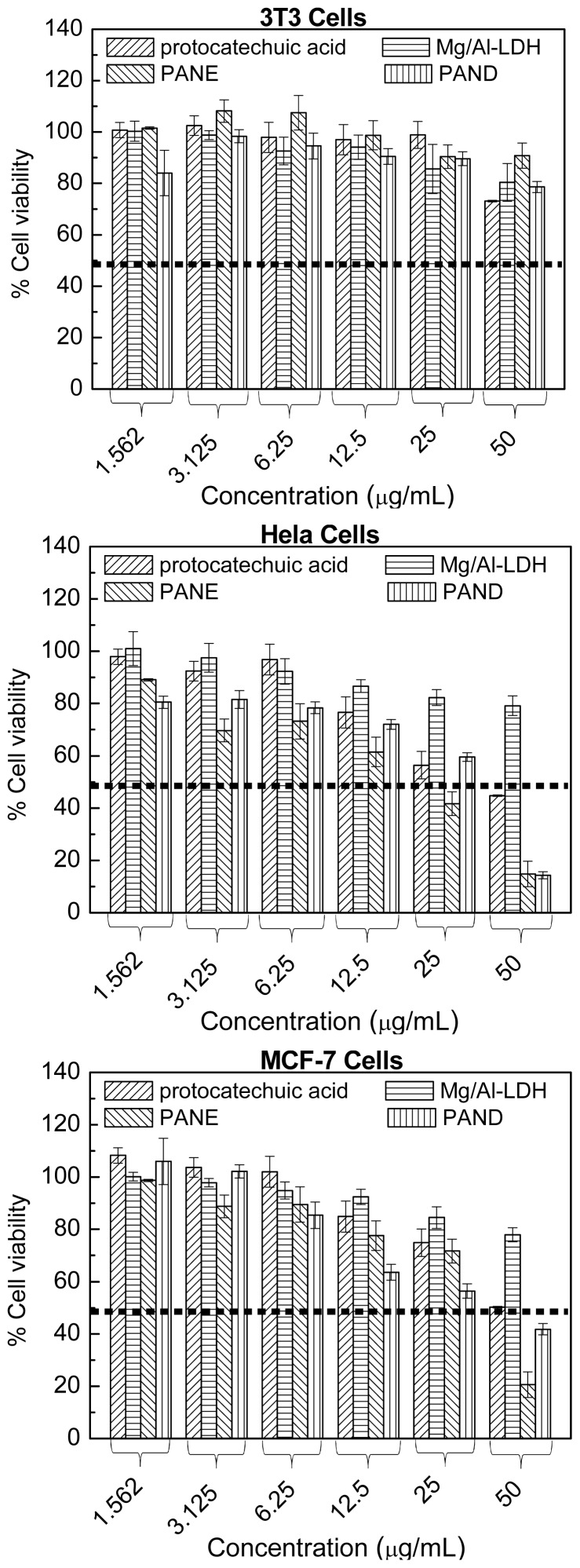
Cell viability (MTT assay) of 3T3, HeLa, and MCF-7 cell lines exposed to various concentrations of protocatechuic acid, PANE, PAND and Mg/Al-LDH (with permission from Dove Press, Auckland, New Zealand).

The cytotoxicity of protocatechuic acid (PA), PANE (PA-Mg/Al-LDH by ion exchange method), PAND (PA-Mg/Al-LDH by direct method), and Mg/Al-LDH (Figure 5) after 72 h incubation at various concentrations in MCF-7 (human breast cancer), HeLa (human cervical cancer), and 3T3 (normal fibroblast) cell lines showed that Mg/Al-LDH was not toxic in both cancer and normal cell lines. In addition, the nanocomposites showed higher suppressive effects in both cancer cell lines than the free drug, without toxic effects in normal fibroblasts cells [61].

The *in vitro* cytotoxicity study of etoposide-LDH nanohybrids, free etoposide, and Mg/Al-LDH up to 40 μg/mL concentration after 48 h of exposure in normal GES-1 cells and MKN45 and SGC-7901 tumor cells showed that the Mg/Al-LDH did not cause any toxic effects in these cells. However, the intercalation process significantly decreased the toxic effect of etoposide on GES-1 normal cells and indicated higher suppressive effects on MKN45 and SGC-7901 cancer cells than unbounded etoposide [75].

The cell viability and bioassay study of pristine Mg/Al-LDH on HCT-116 (human colon carcinoma) cells showed no significant cytotoxic effect after 24, 48, and 72 h exposure at the same concentrations. The LDH-MTX nanohybrid exhibited effective inhibition on HCT-116 cell proliferation compared to free methotrexate and the IC_50_ of LDH-MTX on HCT-116 cell lines at 48 h exposure times was the same value as the IC_50_ of the free drug at 72 h [78].

The MTT assay study of ellagic acid nanohybrid, ellagic acid, and ZnO on rat hepatocytes cells at different exposure times and 25 μg/mL showed that the ellagic acid nanohybrid and ZnO had mild toxic effects on rat hepatocytes cells comparable to the free ellagic acid. Therefore, the ellagic acid nanohybrid could potentially be used as an anticancer agent in cancer treatment [65].

*In vitro* bioassay studies of combinations of hippuric acid, hippuric acid nanohybrid, or zinc layered hydroxide with tamoxifen in HepG2 (human liver carcinoma) cells at 24, 42, and 72 h incubation times and various concentrations indicated that tamoxifen alone was more toxic to HepG2 cells compared to treatment with combinations of hippuric acid and tamoxifen or ZLH with tamoxifen. However, combination of the nanohybid with tamoxifen showed the highest suppressive effect on HepG2 cells, with an IC_50_ value of 0.35 compared with tamoxifen alone, combination of hippuric acid with tamoxifen, or ZLH with tamoxifen [63].

**Figure 6.**
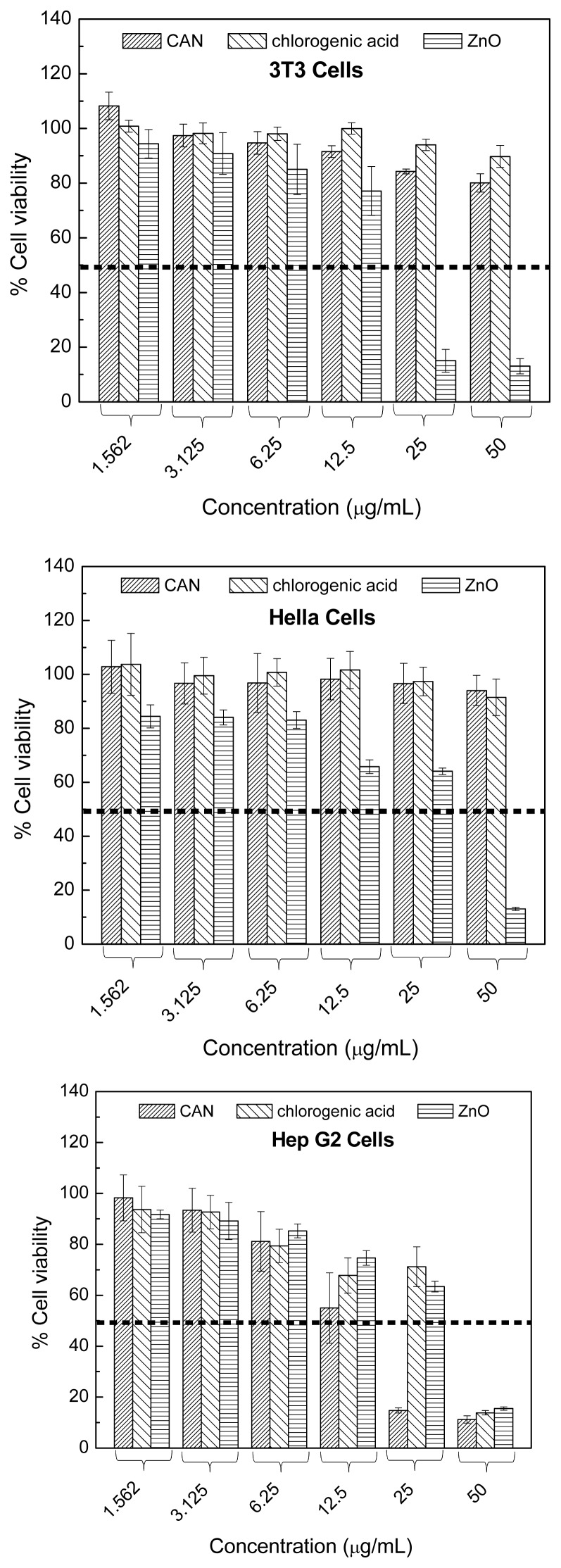

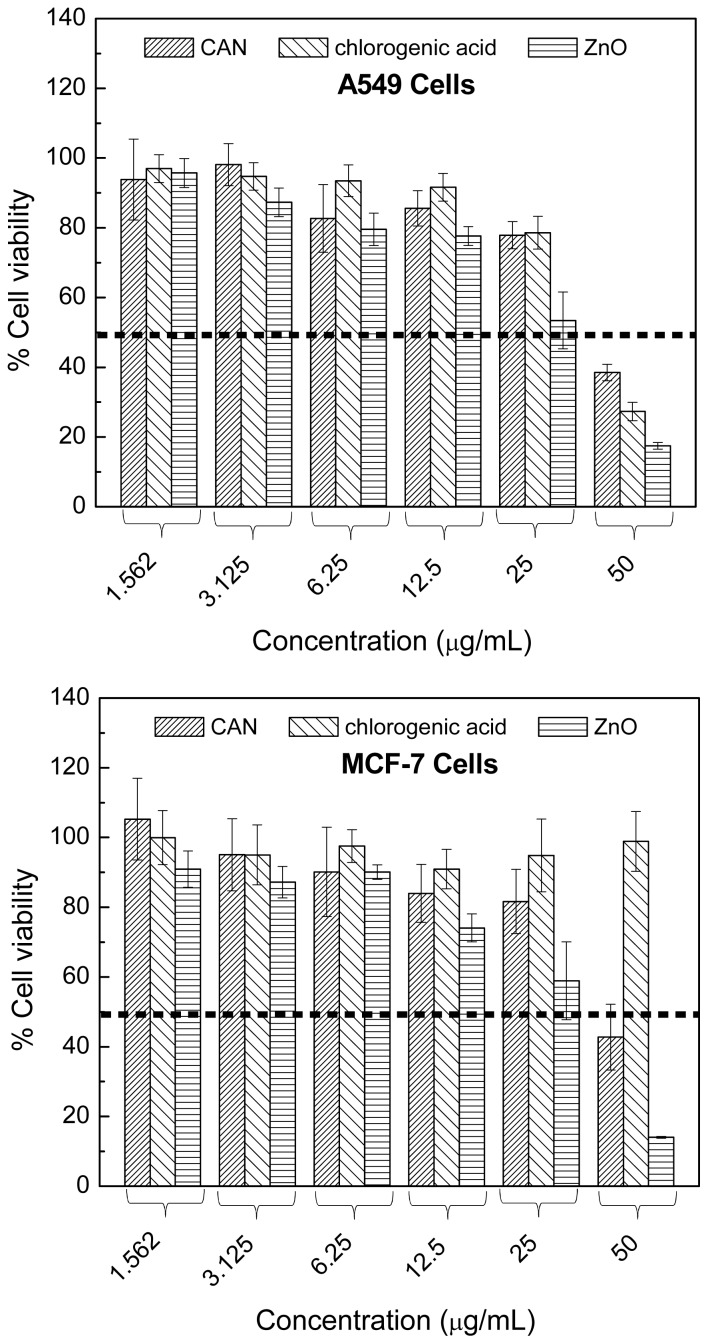
Cell viability (MTT assay) of 3T3, HeLa, MCF-7, A549, and Hep G2 cell lines exposed to various concentrations of chlorogenic acid nanohybrid, free chlorogenic acid and ZnO. (With permission from American Scientific Publisher, Valencia, CA, USA).

*In vitro* cytotoxicity and antitumor assay studies of ZnO, chlorogenic acid nanohybrid, and free chlorogenic acid on 3T3 cells and different types of cancer cells, *i.e.*, MCF-7, HeLa, HepG2,and A549 cell lines (Figure 6), after 72 h incubation at various concentrations indicated that ZnO had a significant toxic effect on both normal and cancer cells at 12.5 and 50 μg/mL concentration, while the chlorogenic acid nanohybrid effectively inhibited cancer cell growth, especially HepG2, in a dose-dependent manner without toxic effect on normal fibroblast cells. Furthermore, chlorogenic acid nanohybrid had a higher suppressive effect on cancer cells, especially HepG2, compared to free chlorogenic acid and it could be established and shows potential for liver cancer therapy (Figure 6) [66].

The cytotoxic effect of cetirizine-Zn/Al-LDH and cetirizine-Mg/Al-LDH nanohybrids was investigated in human Chang liver cells. Both nanohybrids did not show any toxic effect up to 1000 μg/mL. However, the cetirizine-Zn/Al-LDH nanohybrid was slightly more toxic than the cetirizine-Mg/Al-LDH nanohybrid at 1000 μg/mL, and the cell viabilities were 74.5% and 91.9% for the cetirizine-Zn/Al-LDH and cetirizine-Mg/Al-LDH nanohybrids, respectively.

The inhibitory properties on histamine release from Zn/Al-LDH, Mg/Al-LDH, free cetirizine, and nanhybrids was evaluated in RBL2H3 (rat basophilic leukemia) cells. It was clearly observed, as the component concentration increased, that the antihistamine release decreased. In addition, Zn/Al-LDH and Mg/Al-LDH did not have a high inhibitory effect on histamine release in RBL2H3 cells, with different inhibitory properties for both compounds. However, inhibition of histamine release of nanohybrids was lower than that of free cetirizine. This could be due to the small amount of cetirizine in nanohybrids and their slow release properties. Moreover, the inhibitory effect of cetirizine-Zn/Al- LDH was larger than cetirizine-Mg/Al-LDH, which might be due to the higher cumulative release of cetirizine-Zn/Al-LDH compared to cetirizine-Mg/Al-LDH [69].

The cellular up take test for the cetirizine-ZLH nanohybrid (CETN) was carried out using normal Chang liver cells and RBL2H3 cells. The cytotoxicity study of CETN and ZLH at various concentrations showed that they decreased cell viability at 500 and 1000 μg/mL. The IC_50_ for CETN and ZLH was 617 and 670 μg/mL, respectively. In addition, ZLH showed a significant effect on histamine release from RBL-2H3 cells. Additionally, the inhibitory effect of the nanohybrid on histamine release was larger than the unbounded cetirizine at 62.5 μg/mL with 56% and 29%, respectively. Moreover, it was clear that zinc oxide could prevent histamine release [70].

The cytotoxicity of free perindopril, PZAC (perindopril-Zn/Al-LDH prepared by direct method), and PZAE (perindopril-Zn/Al-LDH prepared by ion exchange method) at 24 h incubation and concentrations up to 1.25 μg/mL was evaluated using normal Chang liver cells. Perindopril and both nanohybrids did not display any toxic effects in Chang cells. Moreover, angiotensin-converting enzyme (ACE) inhibition activity by both nanohybrids was found to be time-dependent. The PZAE nanohybrid showed higher ACE inhibition compared to PZAC, which can be explained by the high percentage of perindopril in PZAE compared to PZAC. However, ACE inhibition activity was not observed in Zn/Al layered double hydroxide [67].

The cellular uptake test of Mg/Al-LDH, perindopril-Mg/Al-LDH nanohybrid, and free perindopril was carried in normal Chang liver cells. Perindopril erbumine, Mg/Al-LDH and, surprisingly, the perindopril-Mg/Al-layered double hydroxide nanohybrid did not show any toxic effect in Chang cells. Furthermore, ACE inhibition activity was observed for Mg/Al-LDH around 5.6% after 90 min exposure time. Additionally, the perindopril-Mg/Al-layered double hydroxide nanohybrid inhibited ACE in time-dependent manner. ACE inhibition activity of the perindopril-Mg/Al-layered double hydroxide nanohybrid was larger than perindopril-Zn/Al-layered double hydroxide nanohybrids [68].

The cytotoxicity study of the cinnamate-ZLH nanocomposite on the cell viability of human dermal fibroblasts showed no significant toxic effects up to 12.5 μg/mL of the resulting nanocomposite. However, the cell viability of human dermal fibroblast significantly decreased when they were exposed to 25 μg/mL and above mentioned concentration of nanohybrid. Furthermore, a decrease in cell viability was observed when exposed to 12.5, 25, and 50 μg/mL of ZnO [71].

The cytotoxicity assay study of the salicylic acid nanohybrid (0.8 mol/L), free salicylic acid, and zinc oxide in African green monkey kidney (Vero-3) cells showed a mild toxic effect on cell viability and decreased cell viability in dose-dependent manner. In addition, ZnO was less toxic compared to the salicylic acid nanohybrid and free salicylic acid [97].

The cellular up take test of 4-amino salicylic acid, ZLH and 4-amino salicylic acid nanocomposite after 24, 42, and 72 h incubation and various concentrations was evaluated using 3T3 normal fibroblast cells. The MTT assay showed that the 4-amino salicylic acid nanocomposite decreased cell viability in a time and concentration-dependent manner [72].

The cytotoxic effect of the levodopa nanocomposite, free levodopa, and Zn/Al-layered double hydroxide was investigated in 3T3 normal fibroblast cells. The nanocomposite induced toxic effects in a dose-dependent manner in the concentration range of 5–150 μg/mL. However, pristine levodopa and Zn/Al-LDH nanocarrier at the same dose displayed a higher toxic effect after 72 h exposure time compared with the levodopa-Zn/Al-layered double hydroxide nanohybrid [73].

The cytotoxicity test of free folic acid, folic acid nanohybrid, and LDH was evaluated using human embryonic kidney 293 cells (HEK293T). All samples, after 48 h incubation at various concentrations, did not indicate any toxic effects on HEK293T cells. Moreover, the nanohybrid did not show toxic effects on HEK293T cells, as the cell viability was over 80% even after 72 h of nanohybrid exposure [77]. In addition, the cytotoxic effect of free folic acid, folic acid nanohybrid and Mg/Al-LDH was investigated in normal 293T cells. The folic acid-LDH nanohybrid, free folic acid and pristine Mg/Al-LDH showed no toxicity in normal 293T cell lines [106].

The cellular uptake and antitumor test of 5-FU, 5-FU-Mg/Al-LDH nanohybrid and Mg/Al-LDH after 72 h incubation was evaluated using Human lung carcinoma (A549), liver carcinoma (Hep1), and (HOS) human osteosarcoma cell lines. The Mg/Al-LDH nanocarrier did not show any toxic effects up to 500 mg/mL on these cancer cells. The 5-FU-Mg/Al-LDH nanohybrid had a higher suppressive effect on the cancer cell lines compared to free 5-FU, whereas the IC_50_ values were approximately 2.5–4.3 fold lower than that of free 5-FU. Moreover, comparison of tumor suppression of 5-FU-Mg/Al-LDH with MTX-LDH on the cell lines revealed that MTX-LDH had a higher tumor suppressive effect than 5-FU-Mg/Al-LDH [115].

## 8. The Effect of Physicochemical Properties on Layered Hydroxide Cytotoxicity

Chemical composition is one of the important factors that affect the cytotoxicity of layered hydroxides. Previous studies have shown that zinc-based nanolayers had higher cytotoxicity than the nanolayers composed of other elements, because [Zn_0.68_Al_0.32_(OH)_2_] (CO_3_)_0.16_·0.1H_2_O was slightly more toxic than [Mg_0.68_Al_0.32_(OH)_2_] (CO_3_)_0.16_·0.1H_2_O, as deduced from the higher release of lactate dehydrogenase and hemolysis. The higher toxic effect of Zn/Al-LDH could be explained by the higher toxicity of Zn^2+^ compared to Mg^2+^, because not only the dissolution of both nanolayers was same, but also their particles were almost the same size [114,116–118].

The effect of particle size on toxicity of layered hydroxides was demonstrated by size-dependent toxic effects on human lung adenocarcinoma epithelial (A549) cells. The nanolayers with a particle size of 50 nm, which was the smallest size, had the highest release of lactate dehydrogenase and IL-8 in comparison to nanolayers with a particle size of 100–300 nm. Furthermore, particles with small size had higher cellular uptake rates than particles with large size. As the small particles have the higher specific surface area than large particles, the biological reactivity of particle is increased, which explains their cytotoxicity [119].

**Figure 7.**
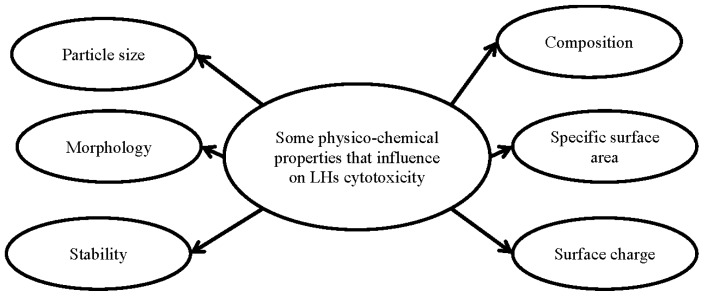
Physicochemical properties of layered hydroxides that affect their cytotoxicity.

Another crucial element, which might have influence on layered hydroxide toxicity, is their chemical stability. The chemical and structural stability of layered hydroxides is affected by the type of incorporated anions into the interlayers and the stabilization by electrostatic interaction between the layers and the anions between the layers. Mg/Al-CO_3_-LDH with high chemical and structural stability and poor solubility in body fluids (low degradability) had significantly higher toxicity in A549 cells than Mg/Al-Cl-LDH with high degradability [120,121] (Figure 7).

## 9. Cellular Uptake Pathway of Layered Hydroxides

Previous research showed that many inorganic nanoparticles can be used for cellular delivery of drugs, but layered hydroxides have been considered as unique nanocarriers for efficient cellular delivery of drugs. This is because of their excellent features, especially the unique surface modification due to their great ion-exchange property, high positive zeta potential (20–50 mV), and layered hydroxides are easily degraded in liposomal media and form ions, compared to other inorganic nanoparticles. In addition, the positively charged layers of layered hydroxides significantly improve the efficient cellular delivery of drugs. The negatively charge membrane of cells prevents drugs with negative charge from entering the cells. By incorporating anionic drugs into the layers of layered hydroxides, these drug-nanohybrids with positively charged surfaces are able to properly enter the cell and enhanced cellular delivery of drugs [45,122,123].

Previous studies showed that the cellular uptake of layered hydroxides occurs through clathrin-dependent endocytosis. Fluorescein isothiocyanate (FITC) was intercalated into the gallery space of LDH with a particle size of 150 nm and hexagonal shape to effectively trace the cellular uptake pathway of LDH in a human bone cancer (osteosarcoma) cell line. The cellular uptake pathway was monitored and observed using immunofluorescence and confocal microscopy. It was observed that the FITC-LDH co-localised with typical proteins related to clathrin-mediated endocytosis, including clathrin heavy chain, dynamin, and EPS15 [124]. In addition, the cellular uptake pathway of LDH via clathrin-mediated endocytosis was confirmed by comparison of fluorescein isothiocyanate-LDH (FITC-LDH) location inside the cell with that of transferrin, a well-known marker for clathrin-mediated endocytosis.

Furthermore, the modification of the layered hydroxide morphology may influence the subcellular distribution. LDH with its hexagonal shape was localized in perinuclear cytoplasm. However, rod shaped LDH was mainly distributed in the nucleus [124].

Moreover, the particle size had a significant effect on the rate of LDH cellular uptake. The previous reports showed that LHs with a particle size of 50 nm, which was the smallest size, had the highest cellular uptake, and followed by 200 ≥ 100 > 350 nm. By increasing the particle size of LDH, the cellular uptake of LDH was decreased. Interestingly, it was found that the cellular uptake of all sizes of layered hydroxides was completed within 15 min [119,125].

## 10. Conclusions

Layered hydroxides with their unique features as nanovehicles have revolutionized the medical science particularly in drug delivery. The incorporation of drugs into layered hydroxides improves the thermal and chemical stability, cell targeting, solubility of drugs, decrease side effects, and resistance of drugs to disease treatment, and increase plasma half-life of drug.

Overall, layered hydroxides show great potential for use as nanocarriers, with the efficient cellular delivery of drugs, that increases the therapeutic efficiency of drugs in the treatment of different diseases.

## Figures and Tables

**Table 1.** Various applications of LHs.

Application	Examples	References
Water treatment	CrO_4_^2−^ was removed from contaminated water by Ca_38_Al_2_(OH)_11.6_Cl_2_(H_2_O)_5.8_. The adsorption during the synthesis was used for removal of Zn^2+^/CrO_4_^2−^ from waste water. Cement, which contained Al_2_O_3_, was mixed with Ca(OH)_2_ to provide Ca/Al and then used to treat the mixture of Zn_2+_/CrO_4_^2−^. The resulting product was a Zn/Al-CrO_4_^2−^ layered double hydroxide.	[25]
	Li-Al-LDH was synthesized through the co-precipitation and homogeneous precipitation methods and used for fluoride removal from water. Li-Al-LDH exhibited high fluoride adsorption capacity.	[26]
	Zn/Al-Cl-LDH was used to remove RB19 [2-(3-(4-Amino-9,10-dihydro-3- sulpho-9,10-dioxoanthracen-4-yl) aminobenzenesulphonyl) vinyl) disodiumsulphate] dye from contaminated water. Zn/Al-Cl-LDH showed excellent RB19 adsorption from the aqueous solution.	[27]

Anticorrosion agent	Intercalation of the corrosion protection agent, benzoate anion into the Zn/Al-LDH enhanced its anti-corrosion property so that the resulting LDH materials significantly decreased corrosion rate in Q235 carbon.	[28]
	P-aminobenzoate (pAB) was intercalated into Mg_2_Al-CO_3_-LDH to produce Mg_2_Al-pAB, which can remarkably reduce corrosion in simulated concrete by decreasing the free chloride concentration in simulated concrete solution through ion-exchange between free chloride anions and pAB anions in the Mg_2_Al-pAB structure.	[29]
	The layered zinc hydroxide with sulphate as the counter anion showed high protective ability and corrosion resistance of steel and iron substrates. The zinc hydroxide sulphate layer is generated by exposing galvanic Zn and Zn-Mn alloys to a freely aerated solution of Na_2_SO_4_.	[30]

Catalyst	Thiamine pyrophosphate-Mg/Al and Thiamine pyrophosphate-Zn/Al-LDH nanocomposites were used as heterogeneous catalysts for decarboxylation of pyruvic acid and enhanced catalytic activity of thiamine pyrophosphate (TPP) due to the incorporation of thiamine pyrophosphate into the interlayer gallery of LDH.	[31]
	Mg/Al-LDH synthesised at a Mg/Al ratio (R) of 2 exhibited higher catalytic efficiency in the conversion of fatty acid methyl esters to monoethanolamides compared to Mg/Al synthesized at R of 3 prepared by the same method.	[32]
	Zinc hydroxide nitrate (Zn_5_(OH)_8_(NO_3_)_2_·2H_2_O) was intercalated with anionic iron porphyrin [Fe(TDFSPP)], and the resulting nanocomposite showed significant catalytic activity for the oxidation of cyclohexane to *tert*-butyl alcohol.	[33]

Flame retardants	Incorporation of acrylonitrile-butadiene-styrene (ABS) resin into Mg/Aland ZnMg/Al-LDHs leads to significant improvement in smoke suppression and reduction in flammability rate.	[34]
	Intercalation of flame retardants, namely ammonium polyphosphate, pentaerythritol, or melamine cyanurate, into Zn/Al-LDH enhanced the fire retardant property of polylactic acid (PLA) and the PLA-FR-Zn/Al-LDH nanocomposite showed higher flame retardant efficiency.	[35]
	Low-density polyethylene (LDPE) has been intercalated into Mg/Al-LDH, which improved flame retardant property of LDPE.	[36]

Sensors and electrodes	Mg/Al-LDH intercalated with cobalt-ethylenediaminetetraacetate (Co(II)-EDTA) complex was used as a chemical/biological sensor for H_2_O_2_ detection and showed great selectivity for H_2_O_2_.	[37]
	Hemin-Fe/Ni-LDH nanocomposite-modified electrodes could accomplish the role of the natural enzyme, peroxidase, and could also be used in H_2_O_2_ detection.	[38]
	LDHs were used for preparation of cathode materials, Li[Co*_x_*Ni*_y_*Mn_1−x−y_]O_2_ for lithium secondary battery applications.	[39]

Other applications	Enhancement of thermal stability and *uv*-absobance of polypropylene (PP) were observed with Mg_3_Al-tartrazine LDH nanocomposite.	[40]
	Enhancement in thermal stability and mechanical properties of thermoplastic polyester elastomers were observed by encapsulation of benzoate into zinc hydroxide nitrate.	[41]
	Uranium ions were removed from aqueous solution using *in situ* grow of a nanohydroxide on magnetic Ca/Al-LDH followed by calcination.	[42]

**Table 2.** Equations used for fitting controlled release profiles of drugs.

Kinetics Models	Equation	Reference
1. Pseudo-first order	ln(*q*_e_ − *q*_t_) = ln *q*_e_ − *kt*	[61–64]
2. Pseudo- second order	*t*/*q*_t_ =1/*kq*_e_^2^ + *t*/*q*_e_	[19,47,61,63,65–74]
3. parabolic diffusion	(1 − *M*_t_/*M*_o_)/*t* = *kt*^−0.5^ + *a*	[61,75–77]
4. modified Freundlich	1 − *M*_t_/*M*_o_ = *kt^a^*	[75]
5. Ritger–Peppas	*X* = *k*(*t* − *R*)*^n^*	[78,79]
6. Bhaskar	ln(1 − *X*) = −1.59(6/*d*_p_)^1.3^ *D*^0.65^*t*^0.65^	[64,80]
7. Higuchi	*X* = *k*(*t* − *R*)^1/2^	[81]
8. Wei bull	*M* = *M*_∞_[1 − *e* − ((*t* − *t*_o_)/*t*_d_)*^β^*]	[82]
9. Korsmeyer-Peppas	*M*_r_/*M*_f_ = *kt^n^* + *a*	[83]
